# Characterization of the Hypercitrullination Reaction in Human Neutrophils and Other Leukocytes

**DOI:** 10.1155/2015/236451

**Published:** 2015-05-19

**Authors:** Yebin Zhou, Tiziana Di Pucchio, Gary P. Sims, Nanette Mittereder, Tomas Mustelin

**Affiliations:** Department of Respiratory, Inflammation, and Autoimmunity, MedImmune LLC, One MedImmune Way, Gaithersburg, MD 20878, USA

## Abstract

Autoantibodies against citrullinated proteins are diagnostic for rheumatoid arthritis. However, the molecular mechanisms driving protein citrullination in patients with rheumatoid arthritis remain poorly understood. Using two independent western blotting methods, we report that agents that trigger a sufficiently large influx of extracellular calcium ions induced a marked citrullination of multiple proteins in human neutrophils, monocytes, and, to a lesser extent, T lymphocytes and natural killer cells, but not B lymphocytes or dendritic cells. This response required 250–1,000 *μ*M extracellular calcium and was prevented by EDTA. Other neutrophil activating stimuli, such as formyl-peptides, GM-CSF, IL-6, IL8, TNF*α*, or phorbol ester, did not induce any detectable increase in protein citrullination, suggesting that receptor-induced calcium mobilization is insufficient to trigger hypercitrullination. We conclude that loss of membrane integrity and subsequent influx of high levels of calcium, which can be triggered by perforin released from cytotoxic cells or complement mediated formation of membrane attack complexes in the joints of rheumatoid arthritis patients, are sufficient to induce extensive protein citrullination in immune cells, notably neutrophils. This mechanism may provide the citrullinated autoantigens that drive autoimmunity in this devastating disease.

## 1. Introduction

Rheumatoid arthritis is a common form of arthritis, second only after osteoarthritis in prevalence, and is still for most patients a severe, painful, and debilitating disease [1]. Histologically, the RA joint is characterized by an expanding and invasive inflammation, which includes T and B cell infiltrates, sometimes well organized into ectopic follicles, as well as macrophages, dendritic cells, and granulocytes [2–4]. Beginning in the earliest stages of RA, the synovial fluid has a particularly high content of neutrophils [2]. Their presence correlates with joint stiffness, changes in the lubricating properties of the synovial fluid [5], and the escalating inflammation of the surrounding joint tissue referred to as synovitis. In addition, there are early indications that neutrophils may be key for the generation of the anti-citrullinated peptide autoantibodies, which are characteristic for RA [6].

The amino acid citrulline arises from arginine by deimination through the action of protein-arginine deiminases (PAD), one of which, PAD4, is particularly highly expressed in neutrophils. Single-nucleotide polymorphisms in the gene for PAD4,* PADI4*, are consistently associated with RA [7, 8] in Asian population and possibly also in Caucasian populations [9], further strengthening the notion that granulocyte-derived PAD4 may be a key player in the citrullination of proteins in the synovial fluid, which subsequently acts as autoantigens to trigger an immune response resulting in anti-citrulline-peptide antibodies [6].

In this paper, we characterize the hypercitrullination reaction, which can be triggered in neutrophils by perforin and the membrane attack complex of activated complement [10]. Our experiments suggest that these triggers, both of which have been suggested to be present in the RA joint, simply act by flooding the cytoplasm of neutrophils with supraphysiological levels of calcium. We also show that varying degrees of hypercitrullination can be induced in many leukocyte lineages by perforin, ionomycin, and Triton-X. Other activating stimuli fail to induce this response, presumably because they do not elevate the intracellular calcium concentrations to the approximately 250 *μ*M required for detectable citrullination to occur.

## 2. Materials and Methods

### 2.1. Isolation of Primary Human Cells

Human blood from healthy volunteers was obtained with informed consent under MedImmune's blood donation program and studies using human cells were performed in accordance with the Institutional Review Board guidelines of MedImmune LLC. Peripheral blood mononuclear cells (PBMC) and neutrophils were isolated from heparin anticoagulated blood from healthy donors on a discontinuous gradient as previously described [11]. Monocytes and T cells were subsequently isolated by negative immunoselection (Stemcell Technologies, negative selection markers for monocytes were CD2, CD3, CD16, CD19, CD20, CD56, CD66b, CD123, and glycophorin A and for T cells were CD14, CD16, CD19, CD20, CD36, CD56, CD66b, CD123, and glycophorin A) and B cells by positive immunoselection (Miltenyi Biotec, positive selection marker CD19) following the manufacturer's instructions with a purity of over 95%. Plasmacytoid dendritic cells (pDCs), myeloid dendritic cells (mDCs), and natural killer cells (NK cells) were isolated by negative immunoselection from fresh leukopacks obtained from healthy donors (Stemcell Technology, negative selection markers for NK cells were CD3, CD4, CD14, CD19, CD20, CD36, CD66b, CD123, HLA-DR, and glycophorin A; pDC and mDC selection markers were not disclosed by manufacturer) following manufacturer's instructions with a purity of over 95%. All cell populations were then washed and resuspended at a density of 10 × 10^6^ cells/mL in RPMI medium with 5 mM HEPES (Invitrogen). To assess the requirements for calcium, dose-responses using 0, 31.25 *μ*M, 62.5 *μ*M, 125 *μ*M, 250 *μ*M, 500 *μ*M, 1 mM, and 2 mM calcium chloride were added to the medium. Subsequent experiments used either 2 mM calcium chloride or 1 mM EDTA.

### 2.2. Treatment of Cells with Ionomycin, Phorbol 12-Myristate 13-Acetate (PMA), Triton-X, or Perforin

A variety of activating conditions were assessed for their capacity to induce citrullination. Cells were activated with a range of doses of either ionomycin (Invitrogen) for different times at 37°C, 100 nM PMA (Invitrogen) for 1 h or 4 h at 37°C, or 0.005% Triton-X for up to 1 h at 37°C. We chose 100 nM of PMA as this concentration is typically used as a standard concentration for inducing neutrophil extracellular trap formation (NETosis). The sublytic concentration of perforin (Enzo Life Sciences) was determined to be 500 ng/mL for human neutrophils in a previous study [10]. We used 150 ng/mL, 250 ng/mL, and 500 ng/mL to treat neutrophils at 37°C for various times (15 min, 30 min, 1 h, and 4 h). All reactions were stopped by adding lithium dodecyl sulfate (LDS) sample buffer (Invitrogen) and boiling. For controls, neutrophils were either immediately boiled in LDS sample buffer after isolation (time 0 control) or incubated for different times at 37°C before boiling in LDS sample buffer.

### 2.3. Gel Electrophoresis and Western Blot

Proteins from an equivalent number of cells (50,000 cells) were separated by gel electrophoresis on 4–12% SDS gel and then transferred to nitrocellulose membrane. For immunoblots with the monoclonal anti-citrulline antibody (clone F95, mouse IgM, EMD Millipore) and HRP conjugated *β*-actin (Sigma-Aldrich), membranes were directly blocked with fish gelatin without chemical modification. Alternatively, membranes were chemically modified as previously described [12], blocked with milk, and detected with anti-modified citrulline antibody (AMC, rabbit polyclonal, kindly provided by Dr. Felipe Andrade, Johns Hopkins University). The primary antibodies were used as follows: anti-modified citrulline antibody (1 : 4,000, 4°C, overnight), monoclonal anti-citrulline F95 antibody (1 : 1,000, 4°C, overnight), and *β*-actin (1 : 40,000, 37°C, 1 h). For *β*-actin, enhanced chemiluminescence (ECL) was directly used for detection. For AMC and F95, HRP conjugated anti-rabbit IgG and anti-mouse IgM were, respectively, used as secondary antibody before ECL detection. Densitometric analysis was performed with ImageJ (NIH).

## 3. Results

### 3.1. Detection of Citrullination and the Hypercitrullination Response

**Figure 1:**
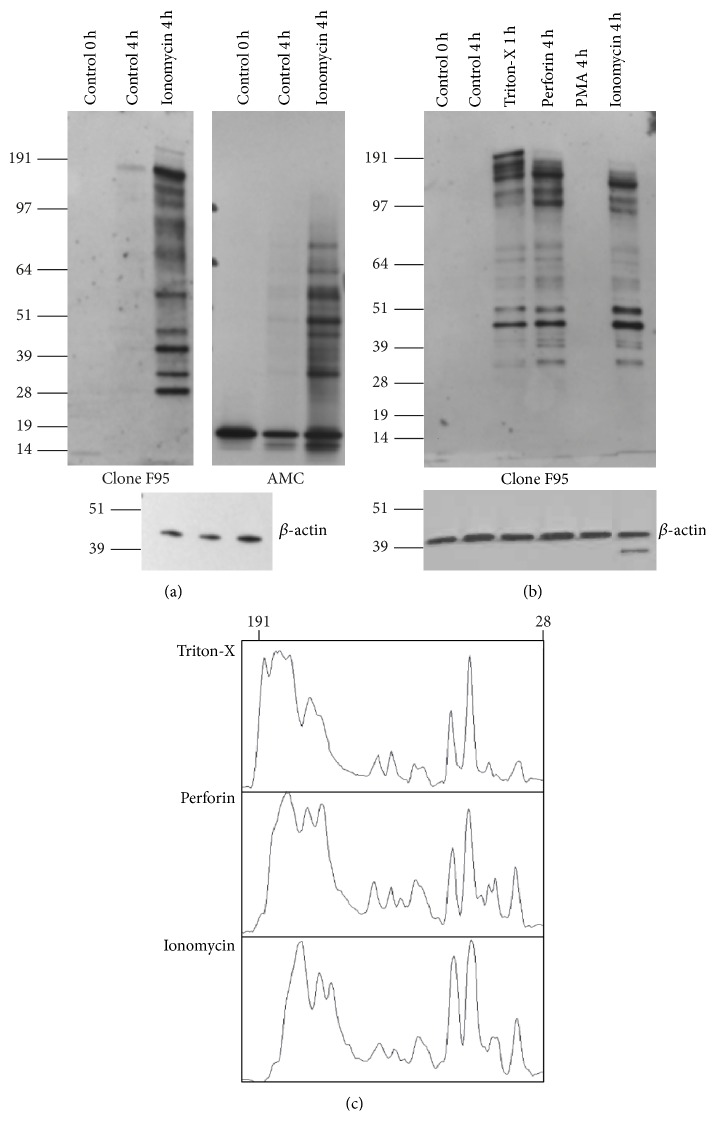
Neutrophil hypercitrullination can be induced by cell membrane lytic conditions. (a) Freshly isolated neutrophils cultured in medium with 2 mM CaCl_2_ alone or in presence of 1 *μ*M ionomycin for 1 or 4 hours. Citrullinated proteins were detected by anti-citrulline antibody clone F95 (left panel) or anti-modified citrulline antibody (AMC) (right panel). Lower panel, immunoblot of the same filter with anti-*β*-actin as a loading control. (b) Freshly isolated neutrophils cultured in medium with 2 mM CaCl_2_ alone or with 1 *μ*M ionomycin, 0.005% Triton-X, or 500 ng/mL perforin for 4 hours and immunoblotted with clone F95 antibody. Lower panel, immunoblot with anti-*β*-actin as a loading control. Similar results were obtained with neutrophils from four additional donors. (c) Densitometric scan of lanes 3, 4, and 6 of the blot in panel (b).

We deployed two independent methods to detect the presence of citrulline residues in cellular proteins. The first used the anti-citrulline antibody F95 from EMD Millipore, which was raised against a polycitrulline peptide coupled to keyhole limpet hemocyanin. The second detection method, which we refer to as “anti-modified citrulline” (AMC), is based on the chemical modification of citrulline residues at low pH to form a ureido group adduct, which is detected by anti-ureido group antibodies [12]. Reactivity to both antibodies was essentially undetectable in untreated human neutrophil samples but increased in a time-dependent manner in cells treated with ionomycin (Figure 1(a)). The band patterns detected by the two detection methods were overlapping but differed in several bands presumably due to differences in the specificities of the antibodies for citrulline residues in different amino acid sequence contexts. The AMC blots also detected a band of Mr ~16 kDa in untreated neutrophils, which may represent histone as this antibody was raised against citrullinated calf histones. In contrast, clone F95 was raised against a synthetic peptide consisting of ten citrulline residues, suggesting that clone F95 may preferentially recognize epitopes with more than one citrulline residue. Nevertheless, since the results generated with the two detection approaches were similar, we mostly used the more convenient F95 antibody for detection of hypercitrullination in subsequent experiments.

Since neutrophil hypercitrullination was reported to be induced by membrane lytic stimuli such as perforin or activated complement [10], we hypothesized that membrane leakage caused by a mild detergent like Triton-X may also lead to hypercitrullination. While cell integrity was not affected by a 4 h treatment with ionomycin or perforin, we did observe that treatment of cells with 0.005% Triton-X for longer than 1 h resulted in significant cell loss, as assessed by Trypan blue exclusion. As shown in Figure 1(b), treatment with 0.005% Triton-X for one hour induced a similar hypercitrullination in neutrophils as the perforin and ionomycin treatments. A densitometry scan (Figure 1(c)) revealed that ionomycin, Triton-X, and perforin treatment resulted in protein citrullination patterns with only subtle differences, which may reflect differences in precise kinetics of triggered calcium influx, as well as the difference in the integrity of cellular lipid bilayers, proteolysis, or other factors. Consistent with the previous study [10], treatment of neutrophils with phorbol ester did not lead to hypercitrullination (Figure 1(b)) at any time point between 15 min and 4 h (data not shown). At the same time, PMA treatment induced a NETosis response confirmed by confocal microcopy (not shown). Consequently, PMA treatment also reduced plasma cell membrane integrity at the 2 and 4 h time points, as observed by DNA staining. Other neutrophil activating stimuli, such as formyl-peptides, GM-CSF, IL-6, IL-8, and TNF*α*, also failed to induce hypercitrullination (data not shown).

### 3.2. Time-Course and Dose-Response of Neutrophil Hypercitrullination

**Figure 2:**
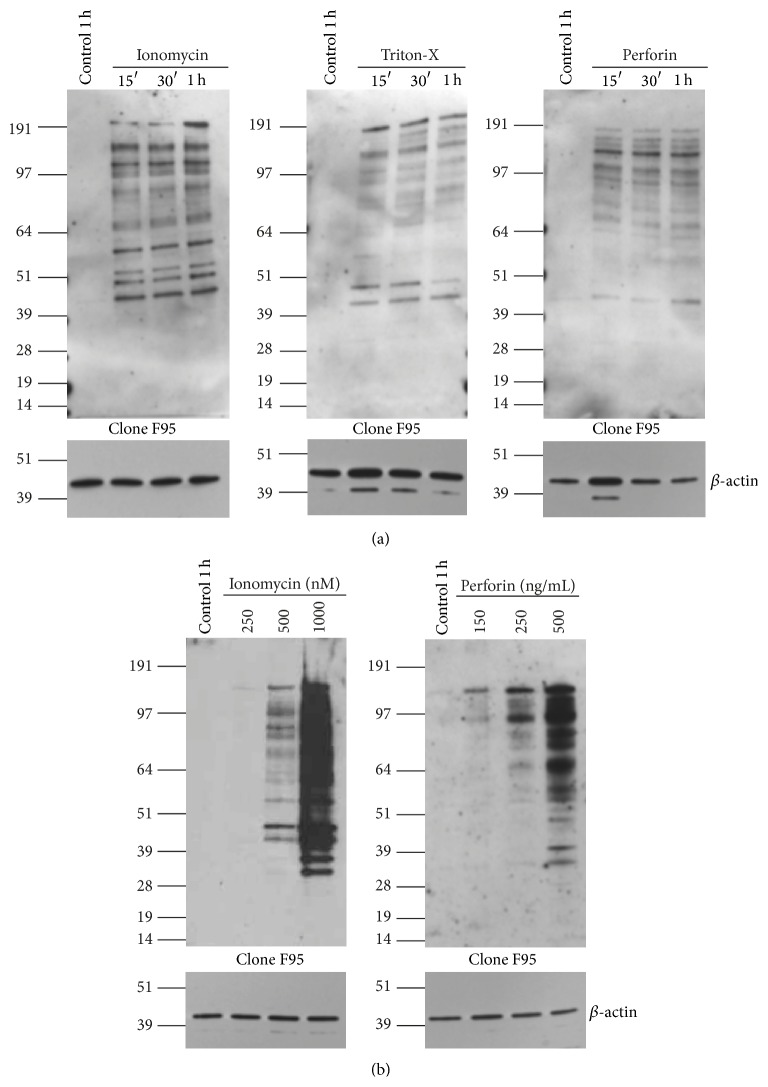
Time-course and dose-response of hypercitrullination in human neutrophils. (a) Isolated neutrophils were cultured in medium with 2 mM CaCl_2_ alone or in the presence of 1 *μ*M ionomycin, 0.005% Triton-X, or 500 ng/mL perforin for the indicated times. Citrullinated proteins were detected by anti-citrulline antibody clone F95 (upper panels) and similar loading of cell lysate was verified with anti-*β*-actin blots of the same filters (lower panels). (b) Isolated neutrophils were cultured in medium with 2 mM CaCl_2_ alone for 1 h or in the presence of the indicated concentrations of ionomycin or perforin for 30 min (upper panels). Anti-*β*-actin blots of the same filters (lower panels). Results are from one donor representative of three different donors.

Time-course experiments with ionomycin, Triton-X, and perforin showed that much of the hypercitrullination reaction occurred within the first 15 min (Figure 2(a)). The dose-responses to ionomycin and perforin showed that 1 *μ*M of the former and 500 ng/mL of the latter were most active (Figure 2(b)), with slight variation from donor to donor in the intensity of the bands. For induction of T cells proliferation, ionomycin is often used at 300 nM, suggesting that the hypercitrullination response in neutrophils requires higher calcium concentrations than those required to promote the proliferative responses in T cells.

### 3.3. Calcium Dependence of Neutrophil Hypercitrullination

**Figure 3:**
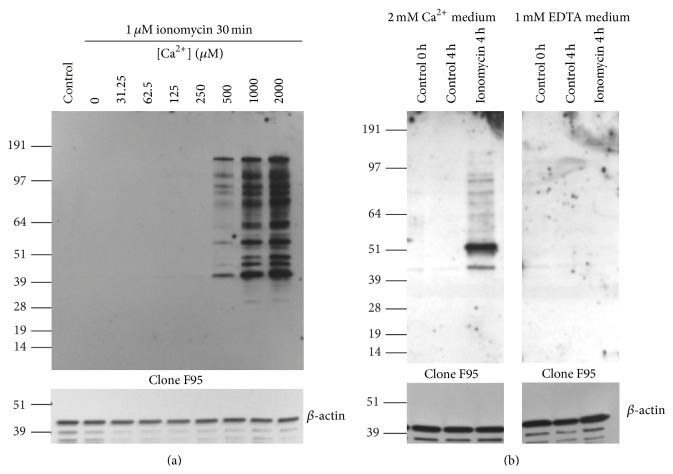
Neutrophil hypercitrullination requires extracellular calcium. (a) Freshly isolated neutrophils were cultured in medium with 2 mM CaCl_2_ alone or in medium with the indicated concentrations of calcium and 1 *μ*M ionomycin for 30 min. Citrullination was detected with the clone F95 antibody (upper panel) and equal loading verified with anti-*β*-actin (lower panel). (b) Neutrophils were incubated in medium with 2 mM CaCl_2_ (left panel) or with 1 mM EDTA (right panel) alone for 0 or 4 hours or with 1 *μ*M ionomycin for 4 h. Citrullination was detected with the F95 antibody (upper panel) and equal loading verified with anti-*β*-actin (lower panel). Results are shown from one donor representative of three different donors.

*In vitro*, the enzymes responsible for protein citrullination require millimolar calcium concentration to be fully active. As in previous studies, we initially used 2 mM Ca^2+^ in the cell culture medium. To test how much calcium was required for the hypercitrullination reaction in human neutrophils, we induced this reaction with ionomycin in the presence of different calcium concentrations (Figure 3(a)). Neutrophils from most donors showed a half-maximal response around 0.5 mM Ca^2+^, but with some heterogeneity among donors with EC_50_ values between 0.25 and 2 mM (data not shown). Notably, hypercitrullination was completely blocked when EDTA was added to the medium (Figure 3(b)).

### 3.4. Hypercitrullination Can Also Be Induced in PBMC, Monocytes, T Cells, and NK Cells

**Figure 4:**
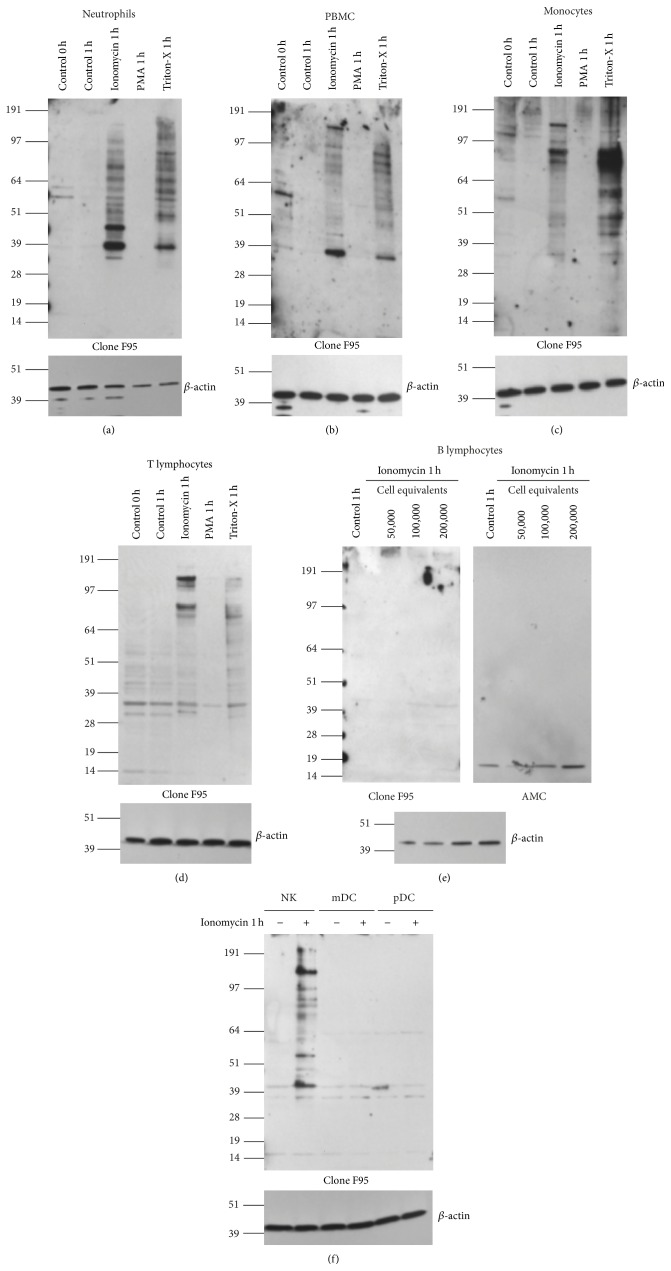
Hypercitrullination in other leukocytes. (a) Neutrophils, (b) PBMC, (c) monocytes, and (d) T cells were isolated from the same donor and cultured with or without 1 *μ*M ionomycin, 100 nM PMA, and 0.005% Triton-X for one hour in medium with 2 mM CaCl_2_. Citrullination was detected with the F95 antibody (upper panel) and equal loading verified with anti-*β*-actin (lower panel). The shown blots are representative of four different donors. (e) B cells were isolated and cultured with or without 1 *μ*M ionomycin in 2 mM CaCl_2_ medium. The indicated number of cell equivalents was loaded and citrullinated proteins were detected by F95 antibody (left panel) or anti-modified citrulline clone antibody (AMC) (right panel). The shown blots are representative of five different donors. (f) NK cells, myeloid dendritic cells (mDC), and plasmacytoid dendritic cells (pDC) were incubated in medium with 2 mM CaCl_2_ medium alone or with 1 *μ*M ionomycin for 1 h, as indicated. Citrullination was detected with the F95 antibody (upper panel) and equal loading verified with anti-*β*-actin (lower panel). The blot is representative of three different donors.

Since neutrophils can induce hypercitrullination, they have been implicated to play a role in the citrullination of proteins detectable in synovium of RA patients. However, the enzymes responsible for citrullination, particularly PAD4 and PAD2, are also expressed in other leukocytes. Therefore, we wanted to determine if hypercitrullination could also be induced in other cells. First, in parallel with neutrophils we tested human blood mononuclear cells (PBMC). As shown in Figure 4, hypercitrullination was induced in both neutrophils (Figure 4(a)) and PBMC (Figure 4(b)) from the same donor. On a cell equivalent basis (50,000 cells), hypercitrullination was less intense in the PBMC samples and the pattern of bands was somewhat different.

Next we isolated additional immune cell populations and determined if they could be induced to undergo hypercitrullination. Monocytes responded to ionomycin with a clear hypercitrullination response (Figure 4(c)), comparable to neutrophils, while T cells responded with a weaker, but readily detectable, hypercitrullination (Figure 4(d)). As estimated by a titration experiment, where neutrophils and T cells were compared side by side (starting with 50,000 cells for both cell types, titrated neutrophil loading down to 1/5, 1/10, 1/100, and 1/500 of T cells), the level of citrullination in T cells was 10–100 times lower than in neutrophils (data not shown). It is also notable that the hypercitrullination reaction was below the detection limit in T cells from some donors.

In contrast to T cells and monocytes, B cells did not show any response in any of our experiments (Figure 4(e)). We tested purified B cells from five different donors with ionomycin treatment and used neutrophils from the same donors as positive controls, and we loaded up to 5 times more cell equivalents per lane. Nevertheless, no hypercitrullination response was detected with either F95 or AMC antibodies (Figure 4(e)). In contrast, NK cells isolated from human blood underwent hypercitrullination upon ionomycin activation (Figure 4(f)), while neither myeloid nor plasmacytoid dendritic cells were able to generate a hypercitrullination response under our conditions (Figure 4(f)).

## 4. Discussion

Studies with patient-derived anti-citrulline autoantibodies have shown that multiple different citrullinated proteins can act as autoantigens in RA patients [13]. Mass spectrometry studies to identify citrullinated proteins in patient samples have also confirmed that numerous citrullinated proteins exist in these samples [14–16], including abundant extracellular proteins such as fibrinogen and fibronectin, as well as intracellular proteins, some of which are expressed primarily in neutrophils (e.g., neutrophil cytosol factor 1). Based on these discoveries, it appears that extensive citrullination of cellular proteins occurs in RA, some of them inside cells like the neutrophil. Nevertheless, it remains to be elucidated whether the rapid hypercitrullination reaction in neutrophils, and as we show here, is instrumental in the pathogenesis of RA. It also remains to be determined if the induction of citrullination in other immune cells plays a role in RA. The presence of extensive citrullination of extracellular proteins also implies that catalytically active PADs gain access to these substrates through a poorly understood process that may involve leakage from damaged cells, perhaps through perforin- or complement membrane attack complex-induced pores. Some extracellular proteins may also enter the cells to be citrullinated intracellularly. Alternatively, PADs may not be released into the extracellular milieu until cells break apart. In our hands, a low dose of the detergent Triton-X induced a strong hypercitrullination response at low concentrations that presumably allow extracellular calcium to enter the cells to activate PADs but do not lyse the cells within the first hour. Further experiments are needed to determine if these conditions also drive citrullination of extracellular proteins.

A key feature of all studied human PADs is their requirement for millimolar concentrations of calcium for catalytic activity* in vitro* [17]. In a previous study [10], perforin and complement membrane attack complexes were shown to cause neutrophil hypercitrullination in medium with 2 mM Ca^2+^, but not in the presence of EDTA, suggesting that abundant influx of calcium was involved. Our experiments confirm these data and extend them to show that submillimolar concentrations of calcium may be sufficient, at least in some donors. While this brings the response more firmly into the physiologically relevant calcium concentration range, it also may suggest that intracellular PADs are subject to somewhat different calcium requirements than the purified PAD4 and PAD2 enzymes in the test tube. Indeed, it has been suggested that PAD4 can citrullinate histones and transcription factors in the nucleus of stem cells [18], under conditions where local calcium concentrations likely are well below 200 nM. Hence, other cellular factors, for example, associated proteins, may exist in the cells to regulate PADs.

PAD4 has been suggested to be necessary for the neutrophil extracellular trap extrusion response, referred to as NETosis [19]. During NETosis, PAD4 was proposed to citrullinate histones to aid in the unwinding of nucleosomal DNA and to be subsequently found on the extruded DNA fibers [19]. The phorbol ester PMA is a strong inducer of NETosis [20]. However, under our experimental conditions, PMA stimulation did not induce hypercitrullination, perhaps because PMA does not induce a calcium response [21]. As in the case of nuclear PAD4 in stem cells, this physiological role of PAD4 in NETosis must be regulated quite differently from the high calcium-induced hypercitrullination reaction, which may well occur only under pathological conditions.

In this paper, we provide more detailed kinetic data on neutrophil hypercitrullination. While earlier studies [22] have documented the kinetics of neutrophil histone citrullination in response to inflammatory stimuli, a more detailed time-course of the neutrophil hypercitrullination response has not, to the best of our knowledge, been reported before. Previous neutrophil hypercitrullination papers have used rather long incubation times and did not report short time points or/and dose-responses to relevant stimuli or extracellular calcium. We found that hypercitrullination occurs within minutes and requires doses of ionomycin or perforin that cause high levels of calcium influx and that it was abolished by EDTA or EGTA (data not shown). By titrating calcium concentrations, we found that neutrophils from most donors showed a half-maximal response around 0.5 mM of extracellular Ca^2+^, but there was some heterogeneity among donors with EC_50_ values between 0.25 and 2 mM. The instances of lower calcium requirements are particularly intriguing because they are well within the physiological Ca^2+^ concentrations present in synovial fluid and tissues. We can only speculate whether the observed individual variations are representative of differences in intact human subjects and, if so, whether they have any relevance for the susceptibility of individuals to citrullination and the development of RA.

PADs expression in leukocytes is not limited to neutrophils. Earlier studies have shown that ionomycin could induce similar hypercitrullination in monocytes [23]. Our study confirms that hypercitrullination readily occurs in neutrophils and monocytes but extends the observation to NK cells and T cells, but not to B cells or dendritic cells. These differences may be caused by cell lineage-specific differences in the expression of different PADs or differential regulation of the activity of PADs in different immune cells. Further work will be required to determine if hypercitrullination is a more universal response in cells to membrane lytic stimuli under physiological calcium conditions.

## 5. Conclusion

Our study shows that a robust hypercitrullination can be induced in human neutrophils within minutes by pore-forming or membrane lytic stimuli in medium with >0.5 mM extracellular calcium. A hypercitrullination response (albeit at much lower levels) can also be induced in monocytes, NK cells, and T cells (at least from some healthy donors), but not in B cells or dendritic cells. Collectively, these cells are the likely source of citrullinated proteins that activated a citrulline-directed immune response in patients with rheumatoid arthritis.
